# Toward a Disruptive “Click-and-Run” 3D Printing Concept for Manufacturing Epidermal Wearable Electrochemical Sensors

**DOI:** 10.1021/acssensors.5c00682

**Published:** 2025-08-07

**Authors:** Daniel Rojas, María Cuartero, Gastón A. Crespo

**Affiliations:** † UCAM-SENS, 16728 Universidad Católica San Antonio de Murcia, UCAM HiTech, Avda. Andres Hernandez Ros 1, Murcia 30107, Spain; ‡ Department of Chemistry, 7655 KTH Royal Institute of Technology, Teknikringen 30, Stockholm SE-114 28, Sweden.

**Keywords:** additive manufacturing, 3D-printed electrochemical sensor, wearable sensors, industry 4.0, epidermal sensors, sweat digitalization

## Abstract

Epidermal wearable sensing is a revolutionary concept with the potential of accomplishing a genuine digital transformation in research fields, such as sports physiology, clinical diagnostics, and health monitoring. The first wearable sweat sensor was reported 15 years ago, and despite the remarkable progress along this period, substantial challenges remain open concerning the complex nature of the manufacturing process. The recent democratization and extensive application of 3D printing technologies have made the automated fabrication of electrochemical sensors feasible, including their integration into complex structures such as microfluidic devices. Nevertheless, to the best of our knowledge, there is no evidence of full 3D printing automation (i.e., all fabrication steps) of an entirely functional epidermal wearable. In this context, we aim to contribute to the community by introducing the concept of “click-and-run” 3D printing, which refers to the complete printing of an epidermal wearable sensor (but not limited to) by just a “click” followed by a “run”. The run refers to the fact that after the click, you “run” away so that no other operations need to be performed, but it also indicates that you can go directly to “run” the experiments after the click. Evidently, this new concept cannot be materialized with traditional 3D printers. Therefore, we share herein how we envision a new generation of 3D printers specifically designed for overcoming the actual issues related to the manufacturing process of wearable sensors. Accordingly, this perspective article is organized as follows: (i) an overview of the advantages of ubiquitous desktop 3D printers and their potential to facilitate click-and-run printing, (ii) a tutorial revision of the main desktop 3D printing techniques and their relationship to manufacture electrochemical sensors, (iii) the rationalization of the required parts for a wearable sensor, (iv) a review of the recent advances and achievements in 3D-printed wearable sensors, and (v) our own description of the new generation of “click-and-run” 3D printers.

## Graphical abstract

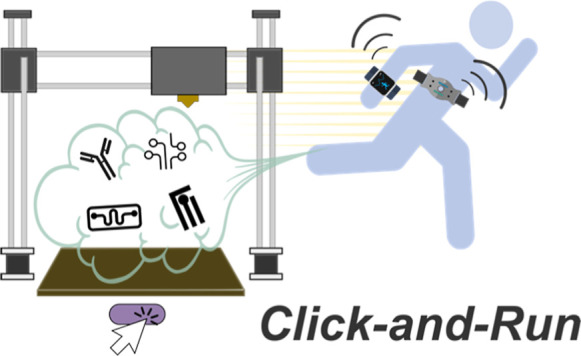

We are currently experiencing a transition to the fourth industrial revolution (Industry 4.0), which is marked by automation, digitalization, and data sharing, resulting in the entire transformation of systems concerning production, management, and governance.^1,2^ Industry 4.0 is recasting almost every global sector, relying on technological advances such as artificial intelligence (AI), augmented reality, industrial Internet of Things, autonomous robotics, big data, cloud computing, additive manufacturing (AM), and smart sensing (e.g., wearables, implantable, in-planta).^3^ This perspective discusses the synergistic relationship among the past, present, and future of the two last technologies in the mentioned list: AM (also known as 3D printing) and smart sensing in the format of wearable electrochemical sensors for sweat. The natural link between these two was first evidenced in reports from the Diamond group, who used simple 3D printing tools to make the holding case (ABS plastic) of a wearable potentiometric sensor for measuring sodium ion in sweat.^4,5^ Since then, 3D printing has been present in the manufacturing process of wearable electrochemical sensors for sweat but, from our modest point of view, without making use of its full potential yet. For example, most of the available reports are based on planar electrode configurations that can in fact be fabricated using 2D techniques (laser-induced graphene, screen printing, inkjet printing), which would indeed provide superior electrochemical performance compared to the 3D approach^6^ (see Table 1).

**Table 1:** List of Works Already Published in the Literature Reporting 3D-Printed Electrochemical Sensors

type of wearable	integrated sensors	level of AM integration	other required techniques	manual steps	accuracy (validation)	comments	ref
patch	temperature	FFF printing of soft TPU for microfluidics and skin interface		manual assembly of electrically conductive parts and electronic components (accelerometer and gyroscope)		body scanning is incorporated for wearable sensor personalized design.	^54^
	strain	conductive TPU used for the strain sensor				no need of tape for skin interfacing	
	gyroscope						
	accelerometer						
patch	sweat rate	skin interface using Polyjet combining rigid and soft materials	roll to roll screen-printing electrodes	assembly of all the layers (electrode, microfluidic, capping, and adhesive layer)	yes (on-body validation		^55^
			roll-to-roll screen-printing of electrodes				
			laser cutting for microfluidic, capping, and adhesive layers				
patch	optical sensing for (Copper, Cl^–^, pH, Glucose)	DLP for microfluidic and optical cuvettes	laser cutting for the microfluidic layer, adhesive, and encapsulation layer.	immobilize assay reagents by drop-castingbonding with the adhesive skin interface	validation of extracted sweat volumes measuring Copper and Cl^–^ using ICP-MS, pH using the pH Tester, and glucose using a fluorospectrometer	single point measurement	^56^
			assembly of all layers	encapsulate top layer			
patch	Cl^–^	DLP for microfluidic channels and capillary burst valves using rigid acrylate-based resin	PDMS fabrication for the epidermal port interface and capping layer	assemble all components (PDMS reservoir capping layer, adhesive gasket, PDMS epidermal port interface, and laser cut adhesive)	validation Cl^–^ concentration of extracted sweat from a second Sweatainer using a chloridometer	integration of capillary burst valves allows multi-time point optical sensing	^57^
			laser cutting for adhesive skin interface				
ring	glucose	multimaterial FFF using TPU and CB-PLA	electroplating of the gold film (−1.0 V for 600 s)		ratio glucose before/after meal similar trend using the ring and glucose meter	pseudo-RE based on CB-PLA not suitable for real applications	^58^
						not (bio)recognition element included	
patch	sweat rate sensor	DIW for the electrode layer (electrode + insulator)	xurography cutting of the microfluidic channel and cover.	assembly of the electrode layer, microfluidic, and cover.	validation using a macroduct with optical sweat rate measurement		^59^
patch	glucose, ethanol, pH, and physical (Temperature and strain)	DIW for printing all the components of the sensor (microfluidics, iontophoresis gel, electrodes and enzyme layer, microsupercapacitors, and electrolyte gel)		assembly of the different layers (microfluidic and iontophoresis, biosensors, and microsupercapacitors) DIW parts	glucose and alcohol sweat sensors were validated by comparing the measurements in sweat with a commercial blood glucose meter and breathalyzer, respectively		^60^
	sweat induction (pilocarpine + iontophoresis)						

In the described context, this perspective article has two primary objectives: first, to stimulate researchers focused on 3D-printed electrochemical sensors to explore wearable sensing opportunities; and second, to inspire those already engaged in wearable sensors to include 3D printing techniques into their workflows. We strongly assert that the association of electrochemists, wearable technology researchers, and emerging 3D printing innovations will refashion the fabrication of wearable sensors, possibly exemplified by the “click-and-run” concept that is being introduced herein. But why are desktop 3D printers likely to lead to a significant advancement in the prototyping of electrochemical wearable sensors and what unique features will they possess? Effectively, it is important to rationalize some of the benefits of shifting the sensor manufacturing paradigm to 3D printing approaches.

Desktop 3D printers offer unprecedented accessibility and decentralization, thus democratizing the manufacturing of electrochemical sensors, formerly confined to specialized central facilities or outsourcing.^7–9^ Decentralized access to 3D printers is especially beneficial for versatility in designing and also for the prototyping and automatized production of wearable devices.

## High Degree of Freedom in Design and Customization

Allows for the incorporation of complex geometry and unique concepts that were previously unattainable with traditional methods of manufacturing.

## The Rapid Prototyping Capability Facilitates Tangible Creations within Hours for Quick and Valuable Feedback

Accelerated iteration of prototypes was achieved by ensuring rapid testing and refinement. By utilization of 3D printers, devices can be fabricated overnight, evaluated in the morning, optimized in the afternoon, and then initiate a new cycle, if needed.

## Cost-Effectiveness in Small-Scale Manufacturing

Contrary to conventional production, which frequently necessitates expensive molds and setups for limited production runs, 3D printing is economically viable for low-volume manufacturing. This permits on-demand production, minimizing the need for inventory, which is especially critical for goods that require specific storage conditions or possess a limited shelf life. In addition, it is convenient for customized products.

## Direct Transition from “Laboratory” to –Fabrication”

Advantageously, the optimized manufacturing capacity can be scaled without changing the production workflow.^10^ This can be accomplished with a straightforward strategy, considering larger printers or using 3D printer farms.

## A Wide Range of Materials with Very Different Properties Can Be Processed

This allows the printing of all components of the wearable sensor platform including insulating materials, conductive elements, flexible structures for body conformity, and even the (bio)­recognition sensing layer.

Considering these features, there is the potential to create a “click-and-run” process wherein a wearable sensor can be produced using a streamlined method that prevents any manual manipulations and is entirely automated via a 3D printer. Thus, the “click-and-run” 3D printing process should encompass four phases, as depicted in Figure 1.

**Figure 1:**
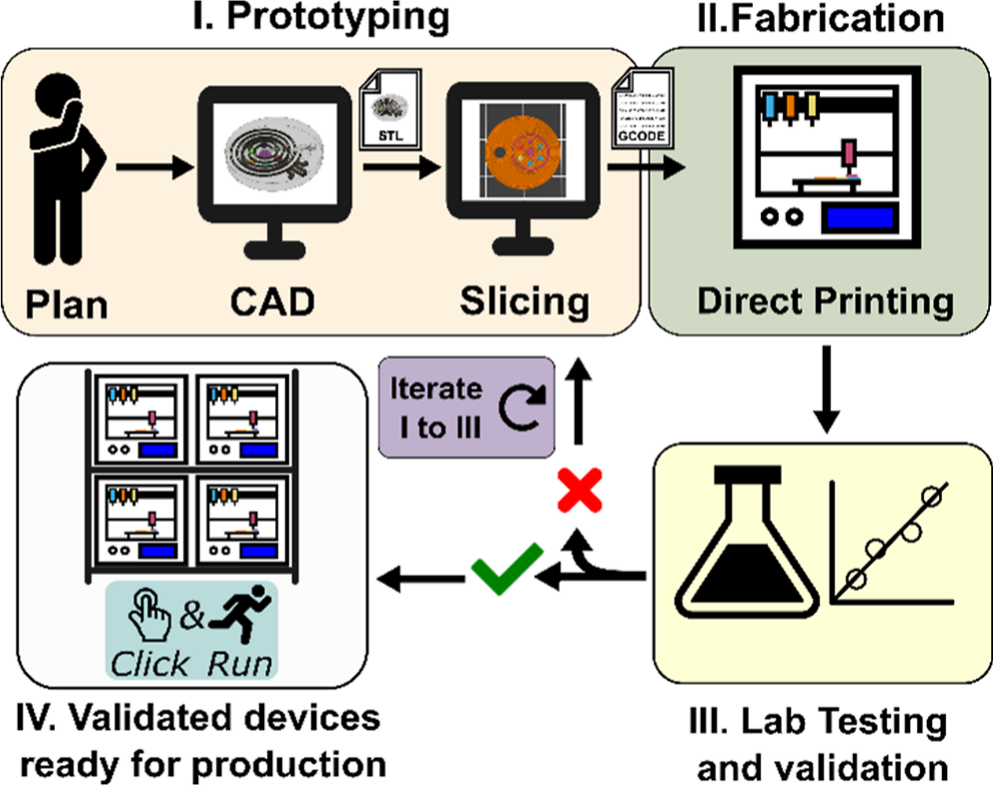
Scheme of the four phases of the “click-and-run” workflow herein proposed for 3D printing wearable epidermal devices.

## Phase I. Prototyping

This phase entails a thorough examination of the physical and chemical characteristics, sensing approach, and specific materials necessary for the desired wearable sensor. Upon finalization of a plausible plan, a three-dimensional model of the object is generated by utilizing a computer-aided design (CAD). After that, a Standard Tessellation Language file is generated, transforming the CAD into a mesh to be processed in Slicer software. Finally, the slicer converts these data into a G-code file containing the motion and operations for the 3D printer.

## Phase II. Printing Process

This phase constitutes the essence of the “click-and-run” 3D printing strategy. It involves the optimization of printing parameters to attain superior print quality, dimensional accuracy, and performance of the printed components.

## Phase III. Systematic Laboratory Testing and Validation

This phase would involve different steps depending on the sensor per se (e.g., dimensional precision, mechanical properties, analytical figures of merit, and accuracy evaluation). In essence, the objective is to guarantee the appropriate functionality of the sensor. If the testing and validation reveal negative outcomes, it is necessary to go along with steps one through three again. In contrast, upon positive validation, the prototype is sent to manufacturing.

## Phase IV. Production

The validated prototype is ready to be automatically produced from a small scale to a high volume. This is achieved through facilities consisting of 3D printer farms or larger printers. Conveniently, owing to the digital nature of the process, the corresponding G-code files can be shared, modified, and executed elsewhere by anyone.

## 3D Printing Techniques: Definition and Milestones for the Fabrication of Electrochemical Sensors

AM, most commonly known as 3D printing, is a broad term that encompasses several technologies categorized by the ISO/ATM 5290013 into seven distinct types: VAT Photopolymerization (VPP), Material Jetting (MJ), Binder Jetting, Material Extrusion (ME), Powder Bed Fusion, Sheet Lamination, and Directed Energy Deposition.^11^ Although all possess applications in many fields, we will focus primarily on ME, VPP, and MJ, which have already demonstrated utility in the development of electrochemical sensors.

### Material Extrusion

ME is an additive manufacturing technique wherein material is selectively extruded in successive layers through a nozzle to construct a three-dimensional object. Fused filament fabrication (FFF) and Direct Ink Writing (DIW) are included in this category. FFF constructs components layer by layer by utilizing filaments composed of thermoplastic materials. The corresponding printer operates by extruding molten material via a nozzle with a specific diameter (0.25–0.8 mm) and depositing it onto a build platform in threads roughly matching the nozzle diameter (Figure 2a). FFF is the technique predominantly used across several industries, generally being the initial method that comes to mind when considering 3D printing. Furthermore, most 3D-printed electrochemical sensors reported up to date have employed FFF owing to its cost-effectiveness, accessibility, capacity for multimaterial production, and the availability of commercial conductive materials.

**Figure 2:**
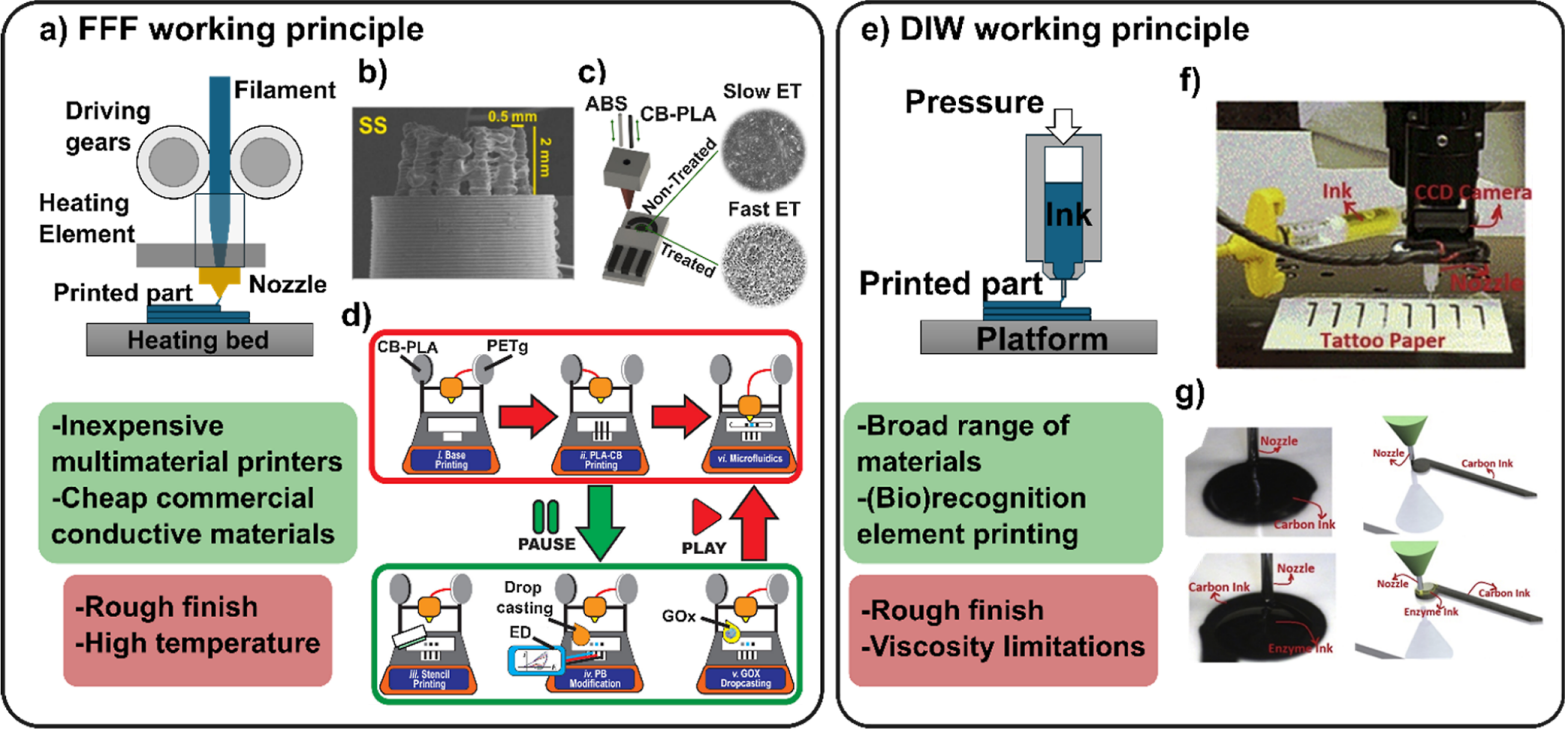
ME techniques represented by FFF and DIW. The green and red squares collect the overall advantages and drawbacks, respectively. (a) General scheme of the working principle of FFF. (b) 3D geometries revealed in FFF-printed electrodes. Reprinted with permission from ref 13. Copyright Elsevier, 2024. (c) Electrochemical cell printed with a multimaterial FFF printer. Treated and nontreated surfaces presenting fast and slow electron transfer (ET), respectively. Reprinted with permission from ref 14. Copyright Elsevier, 2021 (d) General scheme of the PPP protocol for the fabrication of biosensors integrated in microfluidic devices using FFF. Reprinted with permission from ref 26. Copyright American Chemical Society, 2023. (e) General scheme of the DIW working principle. (f) Real pictures and (g) schematics of the DIW process used for the fabrication of the glucose biosensor proposed by Neseai et al. Reprinted with permission from ref 31. Copyright Elsevier, 2018.

Common thermoplastics considered in FFF include polylactic acid (PLA), poly­(ethylene terephthalate glycol) (PETG), and acrylonitrile butadiene styrene (ABS). Then, to produce conductive filaments (e.g., to prepare the parts involving electrodes and connections), thermo-plastics are infused with various carbon allotropes, including carbon black or carbon nanotubes.^12^ And these are essentially the base of any filament, commercially available or custom-made, with most literature taking advantage of commercially available filaments to build electrodes due to their simplicity.

Hussain et al. have recently illustrated the high design versatility offered by 3D printing, somehow abandoning the traditional flat configuration and proposing “skyscraper” electrodes, an image of which is provided in Figure 2b. Certainly, this new design revealed superior analytical performance compared to traditional flat electrodes, i.e., enlarged surfaced area leads to enhanced sensitivity (demonstrated for the case of tumor necrosis factor alpha detection in feces).^13^ Silva-Neto et al. demonstrated how a three-electrode system can be fully 3D printed using both insulating (ABS) and conducting (CB-PLA) filaments, being readily for basic electrochemistry measurements after activation (Figure 2c).^14^ A slow electron transfer was initially observed, especially for inner-sphere redox probes. However, after activation, the electrodes showed an enhanced fast electron transfer toward ferro/ferricyanide couple and paracetamol.

It is noteworthy that the reusability and remanufacturability to produce new sensors have recently been proved using FFF.^15^ In addition to the possibility of remanufacturing sensors, it is also possible to create biodegradable 3D-printed cellulose-based fungal electrodes.^16^ Both of the features are indeed desirable features toward the sustainability of wearable sensors.

Among the flexible FFF-printed electrodes recently reported, Baluchova et al. developed a procedure to integrate boron-doped diamond microparticles and carbon nanotubes (CNTs) within a flexible polymer, thermoplastic polyurethane (TPU), demonstrating both the electroactivity of the filament and flexibility.^17^ Oliveira et al. developed a flexible electrode using a combination of CB and TPU, achieving an ideal balance between flexibility, printability, conductivity, and electrochemical performance by tuning the CB/TPU ratio.^18^ The electrodes were applied for the simultaneous detection of dopamine, uric acid, and nitrite in urine.

To mitigate the ohmic drop, and improving hence the electrochemical output, investigations were focused on developing new filament compositions with large loadings of conductive materials compared to commercial formulations.^12,19^ Also, the integration of graphite/Au nanoparticle composites has been proposed.^20^ Despite the improvement in the conductivity, it remains critical to activate the electrode surface to attain a high electron transfer rate in the electrochemical system. Importantly, to achieve the “click-and-run” philosophy, the activation and indeed any post-treatment step must be automatized, avoided, or not required for the final application. In this context, our group demonstrated the suitability of 3D-printed electrodes for potentiometric sensing without requiring any postprocessing.^21^ Also, we took advantage of 3D printing to create a sort of well surrounding the electrode to template the ion-selective membrane, which improves the reproducibility of the response and avoids the typical water layer effect in solid-contact membrane-based electrodes. This was achieved by fusing the electrode and membrane material, enhancing the sealing of the electrode–membrane interface.

Another aspect to point out is that FFF possesses the capacity to produce complex devices, such as monolithic microfluidics that integrate in turn the electrodes.^22,23^ While useful for some applications, the electroactivity of the 3D-printed electrodes is not adequate for electrochemical reactions that are more complex than outer sphere electron transfer. Recent advances reported by Hernández-Rodríguez et al. have innovated in this direction, allowing the activation of the electrodes when embedded in the microchannel to enhance the electron transfer rate of certain electrochemical reactions.^24^ This was achieved not only by eliminating the outermost layer of the insulator material (PLA) via acetone or NaOH treatment (i.e., solvent-based activation) but also using an electrochemical treatment. Overall, the activations published up to the time of writing have generally consisted of chemical or electrochemical treatments (not optimized to occur within a microfluidic channel) and/or surface mechanical polishing (being hard to translate to the microfluidic case).

Performing additional tasks (e.g., activation and surface modification) on an electrode while it is being produced offers new possibilities to streamline the fabrication of 3D-printed devices. This concept was coined by Pinger et al., naming it Print-Pause-Print (PPP). In essence, the 3D printer was paused to incorporate a dialysis membrane in a space dedicated to it in a 3D-printed device.^25^ The membrane holder was printed, and then, the process is paused at a certain height to accommodate the membrane, being finally resumed. The device was demonstrated to perform equilibrium dialysis experiments to measure the binding affinity of Zn^2+^ to human serum albumin. In contrast to commercially available devices, the proposed device is fully customizable and allows the user to select any membrane to perform the dialysis experiment.

Later, this concept was translated by Hernández-Rodríguez et al. to facilitate not only the activation of 3D-printed electrodes but also other processes.^26^ Briefly, as illustrated in Figure 2d, various electrode modifications can be performed once the printing process is paused: screen-printing to cover the electrode with carbon ink (activation of working electrode) or Ag/AgCl ink to form the reference electrode, electrodeposition of (bio)­sensor transducer (i.e., Prussian-Blue), as well as the drop-casting of the biorecognition element (i.e., glucose oxidase). After that, the printing is resumed, and the modified electrodes remain embedded in the channel forming a monolithic microfluidic device. The potential of the PPP approach is especially realized for the preparation of biosensors, because directly using FFF printing for (bio)­recognition elements is not straightforward. This is likely due to the elevated temperature attained during the extrusion process (>190 °C) and the significant shear stress, which can compromise the integrity of the (bio)­recognition element.

DIW is a printing technique that bears a strong resemblance to its FFF counterpart, with one key distinction: in DIW, the printing material takes the form of a viscous ink, as opposed to the filament utilized in FFF.^27^ The ink, stored within a cartridge, is extruded through a nozzle after the application of pressure, which can be facilitated by a pneumatic pump, hydraulic piston, or motor-driven screw, as schematized in Figure 2e. One of the main challenges in DIW is the compounding of the inks because it needs to meet very specific rheological criteria to achieve a successful extrusion. Under pressure application, the ink displays liquid-like behavior (shear thinning); whereas upon pressure release, as it is extruded through the nozzle, it swiftly transforms back into a solid-like state, retaining its shape until complete solidification.^28^ Notably, the utilization of inks with ideal rheological behavior is a viable option, although it necessitates the implementation of a curing system to instantaneously solidify the material and preserve the integrity of the printed features. Common strategies followed for the solidification of extruded inks include temperature application, photopolymerization, and chemical cross-linking.^29^

DIW has the capability for facile multimaterial printing through the integration of multiple printing heads, analogous to the FFF method. Then, in contrast to FFF, DIW has not only demonstrated the capacity to print not only conductive inks but also inks containing enzymes for glucose and glutamate sensing.^30,31^ The approach used for such a purpose is presented in Figure 2f (real picture of the experimental setup) and Figure 2g (image and scheme of the process). It can be observed how both the carbon ink and the enzyme ink are extruded to form the different layers of the biosensor. In addition, DIW has demonstrated the competence of printing biocompatible cathodes for implantable biofuel cells:

Autopsy and tissues analysis after 1 and 3 months of implantation in rats did not reveal the presence of severe inflammatory reactions.^32^

### Vat Photopolymerization

VPP is a process that utilizes light-activated polymerization to create 3D objects by selectively curing a liquid resin contained in a vat. The curing is performed in areas that are exposed to light, resulting in a solid part. The approach for the delivery of light to the printing layer determines the technique used. Accordingly, there are three distinct methods: stereolithography (SLA), digital light processing (DLP), and masked stereolithography (MSLA), as shown in Figure 3a. SLA is the foundational technology of VPP, developed by Charles Hull in the inaugural commercial 3D printer in 1988.^33^ In this technology, a UV laser photocures the various layers of resin on the build plate. The printing process commences with positioning of the build plate at a specific layer height, corresponding to the focal point of the laser. Then, to achieve different shapes, the laser beam is focused using a set of mirrors, known as galvos, following a layer vector scan (voxel-wise) approach: the UV light selectively cures the resin voxel by voxel, thereby creating the desired shape. The process is repeated in the subsequent layers to create the final shape.

**Figure 3:**
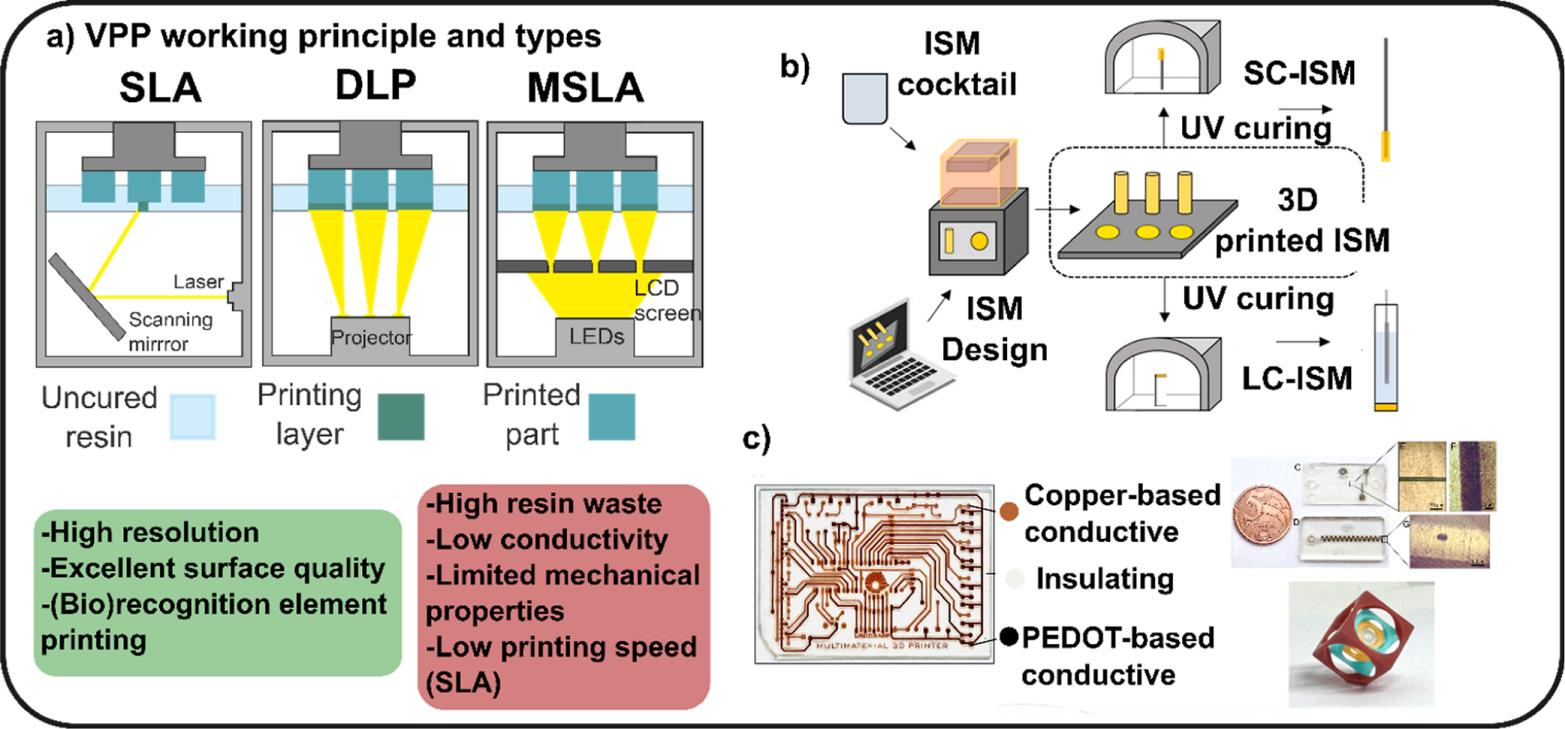
(a) Schematics of the different light configurations used in VPP: stereolithography (SLA), digital light processing (DLP), and mask stereolithography (MSLA). The green and red squares collect the overall advantages and drawbacks, respectively. (b) VPP process developed for the creation of ion-selective membranes using commercial resins. Different shapes are printed, and the concept is demonstrated in liquid contact and solid contact formats. Reprinted with permission from ref 36. Copyright American Chemical Society, 2021. (c) VPP multimaterial printing using copper and PEDOT doped resins. The process was used to obtain complex geometries and microfluidic devices. Reprinted with permission from ref 38. Copyright Royal Society of Chemistry, 2023.

The advent of more advanced optical systems, such as digital micromirrors, led to the development of the DLP. This technology enables the delivery of light across an entire layer simultaneously, reducing hence the printing time while maintaining a comparable resolution as that of SLA. MSLA bears a notable similarity to DLP, with the primary distinction being the projection method. In MSLA, light is projected from an array of LED elements dispersed across the built plate, whereas in DLP, light is projected from a single point. In the MSLA configuration, an LCD screen is used to mask the light. As a consequence, several factors may limit its resolution, including the pixel size of the LCD screen, the collimation of the light, and the uniformity of the light source, preventing its application for high-resolution needs.^34^ Recent studies have demonstrated that resolutions in the hundreds of microns can be attained in microfluidic devices using inexpensive LCD printers (less than €500), which is sufficient for wearable applications.^35^

VPP enables the incorporation of (bio)­recognition elements in sensors. Glasco et al. demonstrated the fabrication of ion-selective membranes for potentiometric sensors by incorporating the membrane components (ion exchanger, plasticizer, and ionophore) into a commercial resin.^36^ As presented in Figure 3b, the membrane can be printed with various shapes and adapted to both liquid and solid contact sensors. The membrane cocktail is prepared by mixing a commercial resin with plasticizer, ion exchanger, and ionophore. Later, the CAD designed shapes are transferred to the printer, and the corresponding membrane is transferred. Once the membrane is obtained, it is postprocessed with isopropanol washing, to eliminate resin excess, and further photocured to ensure the total cross-linking of the resin. Beyond ionophores, other (bio)­receptors, e.g., molecularly imprinted polymers, have been printed using this technique.^37^ Surely, this method is of interest to be translated to wearable electrochemical sensors.

A drawback of VPP may arise when implementing a multimaterial strategy since no commercial solutions are available to the authors’ knowledge. In this direction, Quero et al. developed a modification of commercially available printers by means of peristaltic pumps and the possibility to incline the resin vat to facilitate autonomous cleaning and resin exchange. The authors fabricated several multimaterial objects (Figure 3c) comprising resins with various properties, including flexibility, rigidity, water solubility, as well as fluorescent, phosphorescent, and conductive features. The conductive resins contain PEDOT or copper nanoparticles and were used toward the integration of different properties within a single object.^38^ Cheng et al. modified a DLP printer to automatize the resin exchange process by adding a spinning printing bed. Following the completion of the printing process with a specific resin type, the printing bed rotates, centrifuging the excess resin and subsequently advancing to the next resin vat.^39^ This method streamlines the washing steps and conserves time by eliminating the necessity for vat cleaning. Despite these advances being promising, the absence of commercially available multimaterial printers can impede the advancement of VPP in the field of electrochemical sensor fabrication.

### Material Jetting

MJ is an additive manufacturing process in which droplets of the building material are selectively deposited. It can be conceptualized as a three-dimensional analogue of inkjet printers commonly used in office settings. Figure 4a shows a scheme of the working principle of the most known MJ technique, polyjet. It consists of jetting a photocurable resin through a nozzle on the printed part which is later cured with a light source incorporated in the jetting head. Importantly, in the instance of polyjet, it facilitates the expeditious fabrication of microfluidic devices, yielding surfaces of exceptional smoothness and transparency.^40^ Nonetheless, for the fabrication of hollow structures as microchannels, printing supports are implemented, which later need to be eliminated to clear the channel via postprocessing steps. This considerably increases the production time by hours, making its implementation difficult in the click-and-run workflow herein proposed.^42^ Aerosol jet printing (AJP) is another prevalent MJ technique that functions through the atomization of liquid ink dispersions (Figure 4b).^41^ AJP utilizes an ultrasonic atomizer to generate an aerosol stream containing the ink when it is mixed with a carrier gas flow, propelling the ink out of the nozzle. A sheath flow maintains the aerosolized column as a tight stream as it exits the nozzle, thereby minimizing clogging and overspray. AJP has demonstrated a high degree of compatibility with conductive materials such as those employed in inkjet printing. For example, Tonello et al. developed 3D structured electrodes combining carbon and Ag/AgCl inks to fully print microstructured sensors.^43^ Figure 4c presents the 3D dimensional electrodes with different patterns that can be achieved using AJP, which demonstrated to enhance the electrochemical sensitivity compared to planar electrodes using the redox probe ferro/ferricyanide. Liu et al. fabricated graphene electrodes with the process illustrated in Figure 4d in which the electrodes are printed and later photonically cured layer by layer. To obtain an amperometric sensor for SARS-CoV-2 detection, a further manual modification of the electrode surface with antibodies was required.^44^

**Figure 4:**
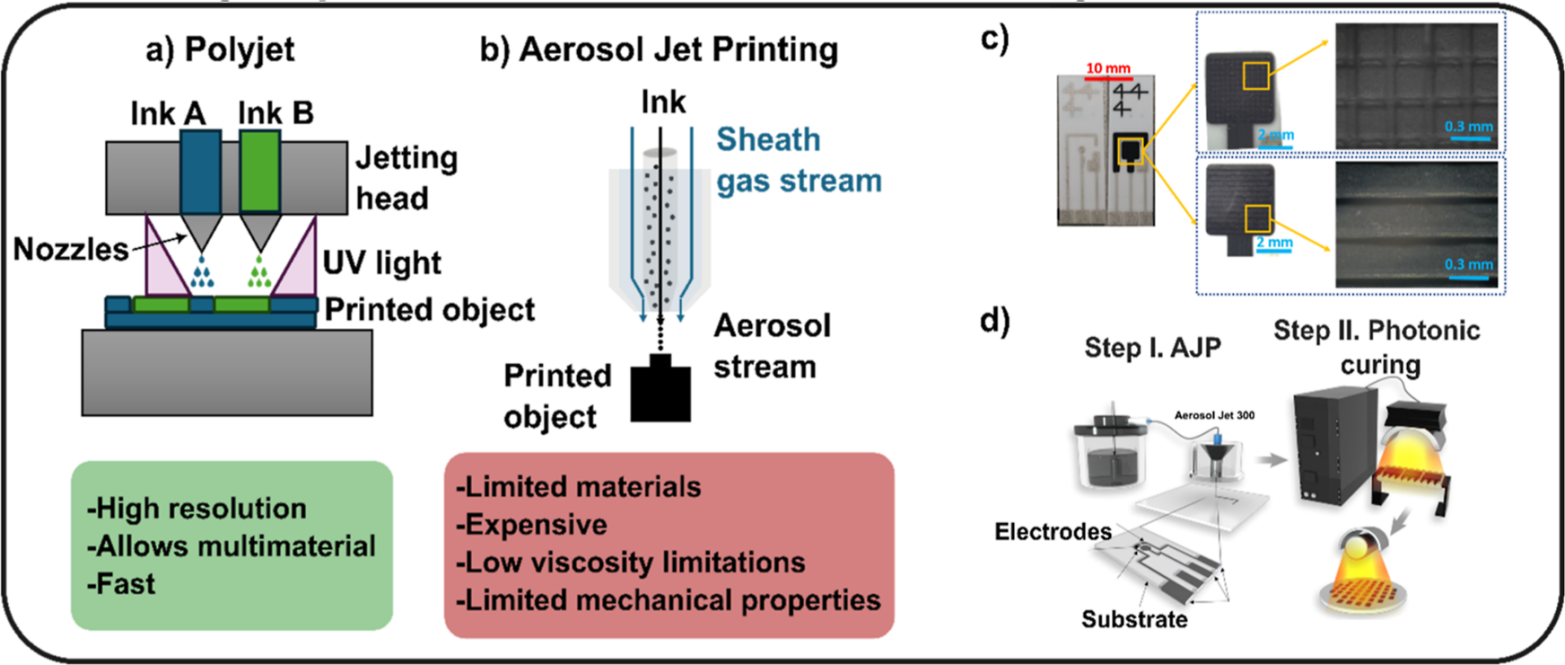
Schematics of printing techniques pertaining to Material Jetting. The green and red squares collect the overall advantages and drawbacks, respectively. (a) Polyjet and (b) AJP working principles. (c) High-resolution 3D features obtained by AJP using different silver-based and carbon-based inks. Reprinted with permission from ref 43. Copyright MDPI, 2021. (d) Schematics of the general process of AJP printing and curing used by Liu et al. Reprinted with permission from ref 44. Copyright American Chemical Society, 2023.

AJP has demonstrated in the previous two examples the capacity to incorporate certain microstructures in the electrodes within the range of hundreds of micrometers, thereby enabling an improvement in the electrochemical signal. However, different curing steps must be carried out between the printing layers. Undoubtedly, the technique is still in its nascent stages with regard to the fabrication of electrochemical sensors. With increasing adoption, it is poised to undergo significant advancements. Indeed, a recent paper by Smith et al. demonstrated the fabrication of carbon-based tracks without the need for any postprocessing, highlighting the potential for further improvements in the field.^45^

## A Glance into Electrochemical Wearable Sensors Ideally Fabricated with 3D Printing Strategies

Let us examine the elements required to fabricate a wearable electrochemical sensor. The objective is to offer a critical perspective on how certain developments in 3D-printed electrochemical sensors may be used in favor of wearable devices. From our point of view, there are five parts that are important to be considered.

(i) Inert components (e.g., the skin interface and microfluidics). These elements maintain the structural integrity of the sensor and manage the sweat sampling and distribution along the wearable. Materials designated as inert components must satisfy specific criteria. In microfluidics, channel dimensions must be a minimum of several hundred microns under active and stimulated sweat conditions. For passive sweating, a larger channel cross-sectional area and a shorter channel length are ideal, with sensors positioned near the inlet.^46^ In instances involving skin interfaces or applications requiring flexibility and stretchability, the mechanical properties of the printed materials must be considered. In such cases, a low Young’s modulus (<100 MPa) along with acceptable stretchability are requisite. Flexible materials or bioadhesive substances can be utilized to secure the skin to the wearable, hence eliminating the need for adhesive tape.^47^
(ii) Physical sensors. Among the options, temperature and sweat rate are two valuable parameters to be used in conjunction to any chemical information. Temperature sensors must be integrated to account for its influence on chemical sensor responses, beyond its physiological interpretation.^48^ Sweat rate or perspiration sensors are required to account for the dilution of analytes in the sweat and to provide complementary data related to sweat loss and dehydration.^49^
(iii) Chemical sensors. These are the key components for the digitalization of chemical data. Depending on the target analyte, certain (bio)­recognition elements and electrochemical techniques will be used, involving different requirements and limitations for the printing of the electrodes and its modification.
(iv) Electronics. There is a growing research and promising commercial solutions for fully 3D-printed electronics, which is especially useful when a customized shape is required, as in the case of wearables.^50^ Nevertheless, the fully 3D-printed electronics will remain out of the scope of this perspective, even being a key component of the wearable sensor.
(v) Sweat stimulation/extraction. For applications relying on passive sweating (e.g., in clinical settings), very small sweat rates (nL/min) limit the application of sweat devices. To overcome this drawback, sweat stimulation electrodes can be integrated in the device. These electrodes rely on chemical stimulation, delivering drugs like pilocarpine or carbachol using iontophoresis,^51^ or thermal stimulation using Joule heaters.^52^ Another recent strategy is based on osmotic sweat extraction, achieved by interfacing the skin with a hydrogel containing a concentrated solute.^53^

Figure 5 depicts the design of an ideal epidermal wearable sweat sensor that we conceived considering the mentioned requirements. In essence, we envision a monolithic platform—defined as a device fabricated in a single piece without the necessity for adhesives, bonding, or assembly—in which the skin interface, chemical sensing, microfluidics for sweat managing, sweat rate and temperature sensor for corrections, and eventual sweat stimulation (for nonsport applications) are contained. This device will enable a direct and resettable connection to the electronics, so the device can be disposed after use. To our knowledge, there is currently no 3D printer capable of producing a monolithic wearable sensor that integrates all the highlighted components. Additionally, considering the “click-and-run” philosophy, different 3D printing techniques shall be integrated into a single workflow since various components with very distinct properties must be incorporated. Such an approach is further discussed in the final section of this perspective article.

**Figure 5:**
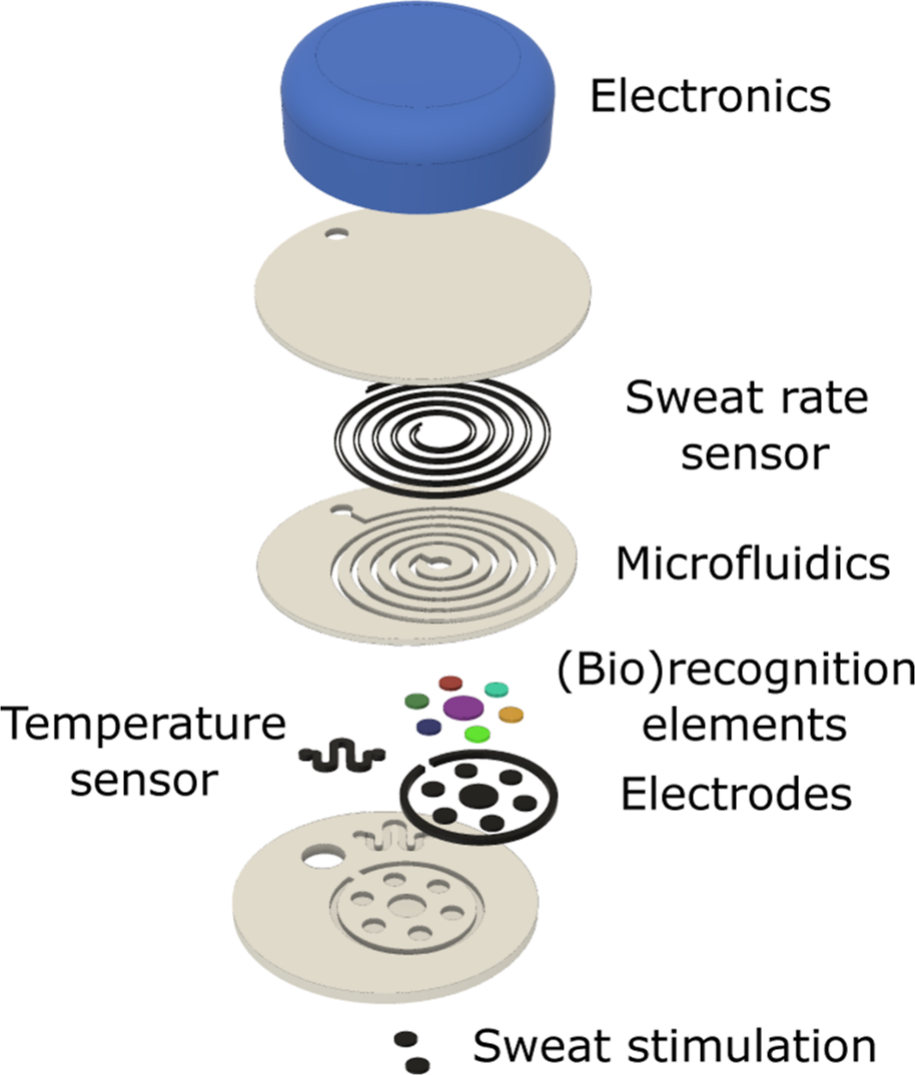
Parts of our idealized 3D-printed epidermal wearable electrochemical patch for sweat analysis. The key components highlighted in the text are assembled in it: (i) sweat stimulation, skin interface, microfluidics; (ii) temperature and sweat rate sensor; (iii) electrodes and (bio)­recognition elements for chemical sensing; (iv) electronics for readout; and (v) sweat stimulation electrodes.

## Recent Advances in 3D-Printed Wearable Sensors for Sweat Analysis

In this section, we scrutinize the role of 3D printing in the development of wearable sensors in recent years. Undoubtedly, a major focus has been dedicated to the adaptation of chemical sensors to specific body parts, taking advantage of the flexibility and versatility of 3D printable materials. For example, Stuart et al. developed certain designs using photogrammetry.^54^ In essence, a mesh of the body shape is obtained, which serves to customize the sensor platform using CAD. Then, it is fabricated by FFF using a flexible material (e.g., thermoplastic polyurethane, TPU). Figure 6a depicts pictures and schemes for all of the steps involved in the design and fabrication process. Notably, a body scan allows the creation of a mesh that conforms to the user’s physiological topology, allowing adhesive-free wearability and optimal sensor placement, which enhance comfort and data accuracy. Also, the design permits the incorporation of nonprinted sensors in a postprocessing step for monitoring physical parameters such as body temperature and strain.

**Figure 6:**
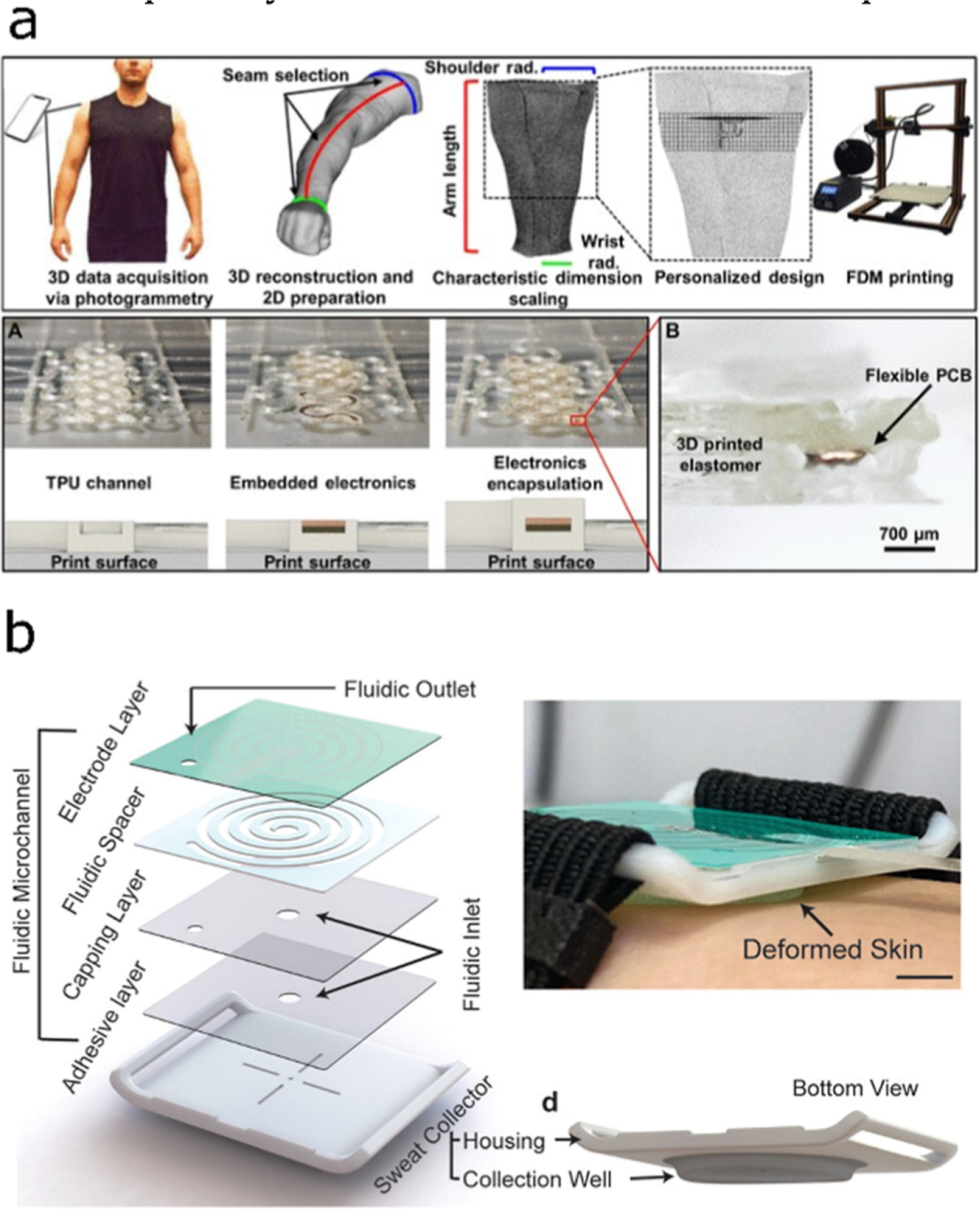
Examples of AM wearable devices found in the literature. (a) FFF-printed wearable device taking advantage of body scanning for wearable conformability. Reprinted with permission from ref 54. Copyright 2021, The American Association for the Advancement of Science. (b) General scheme of the parts of a sweat rate sensor able to avoid the use of adhesive tape for skin adhesion by incorporating 3D design and multimaterial Polyjet printing. Reprinted with permission from ref 55. Copyright 2023, Wiley-VCH GmbH.

Similarly, Dautta et al. integrated a 3D-printed sweat collector using Polyjet technology, combining rigid and soft materials (Figure 6b).^55^ The sensor was designed with a concave surface that was strapped onto the skin to form an effective seal that prevents sweat leakage. Effectively, no adhesive tape was needed in this approach. The collector was further interfaced with laser-engraved microchannels with embedded electrodes for long-term monitoring of the local sweat rate.

Interestingly, despite being beyond the main scope of this perspective, great advances in integrating complete 3D microfluidic systems with optical detection have been achieved. For example, Yang et al. presented a microfluidic system with embedded 3D-printed microcuvettes for multiplexed optical analysis of sweat.^56^ The dimensions of the microcuvettes were demonstrated to be precisely controlled, avoiding any deformation by using rigid materials for the optical path to remain constant, even when the subject who worn the wearable is practicing exercise. For that, the rigid microcuvettes are later encapsulated in a multilayer flexible material, allowing them to be worn on the skin. It was shown that several biomarkers (copper, chloride, pH, and glucose) can be determined in sweat during sauna and cycling. Figure 7a displays the fabrication process involving the 3D printing, washing steps, the immobilization of the different sensing dyes, as well as the encapsulation of the device.

**Figure 7:**
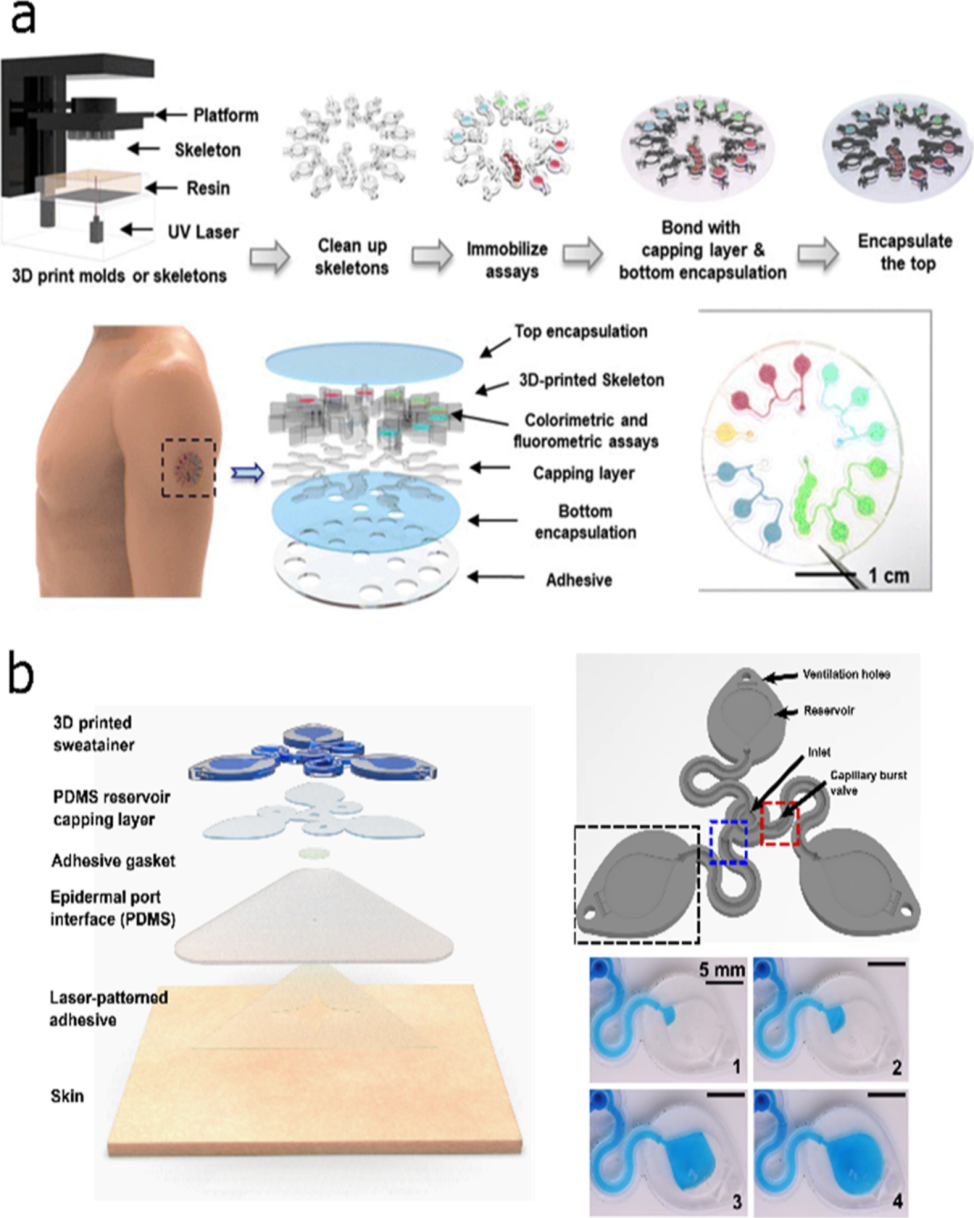
(a) Epidermal optical wearable sensor incorporating soft and hard materials as well as multi-ion detection. Reprinted from ref 56. Copyright 2023, Royal Society of Chemistry. (b) Multilayer scheme and working principle of the Sweatainer device and close view of the bursting valves to collect sweat at different sampling times. Reprinted from ref 57. Copyright 2023, The American Association for the Advancement of Science.

Wu et al. presented the “Sweatainer system”, which allows the collection of multiple sweat samples at different times, overcoming the limitations of current single-point optical wearable devices.^57^ The Sweatainer is fabricated using DLP technology, giving rise to optically transparent devices with channel and valve feature sizes below 100 μm. This high resolution enables the fabrication of capillary burst valves that can control any fluid flow based on pressure thresholds. These valves are designed to prevent fluid flow until the pressure exceeds a certain level corresponding to a fixed amount of sweat accumulated in the device. At this pressure, the valve opens, allowing the fluid to fill the reservoir, which contains in turn a Cl^–^ sensitive dye for sweat analysis (Figure 7b). This work highlights how the integration of complex microfluidic designs into wearable technology can be achieved through AM technology.

Katseli et al. reported an approach to provide electrochemical detection using multimaterial FFF.^58^ A ring composed of flexible TPU and CB-PLA as the conductive part was proposed (Figure 8a). The RE and CE were directly the CB-PLA material, while the WE needed further modification. This latter was postprocessed to be covered with a gold film by electroplating to make the electrode sensitive to glucose.

**Figure 8:**
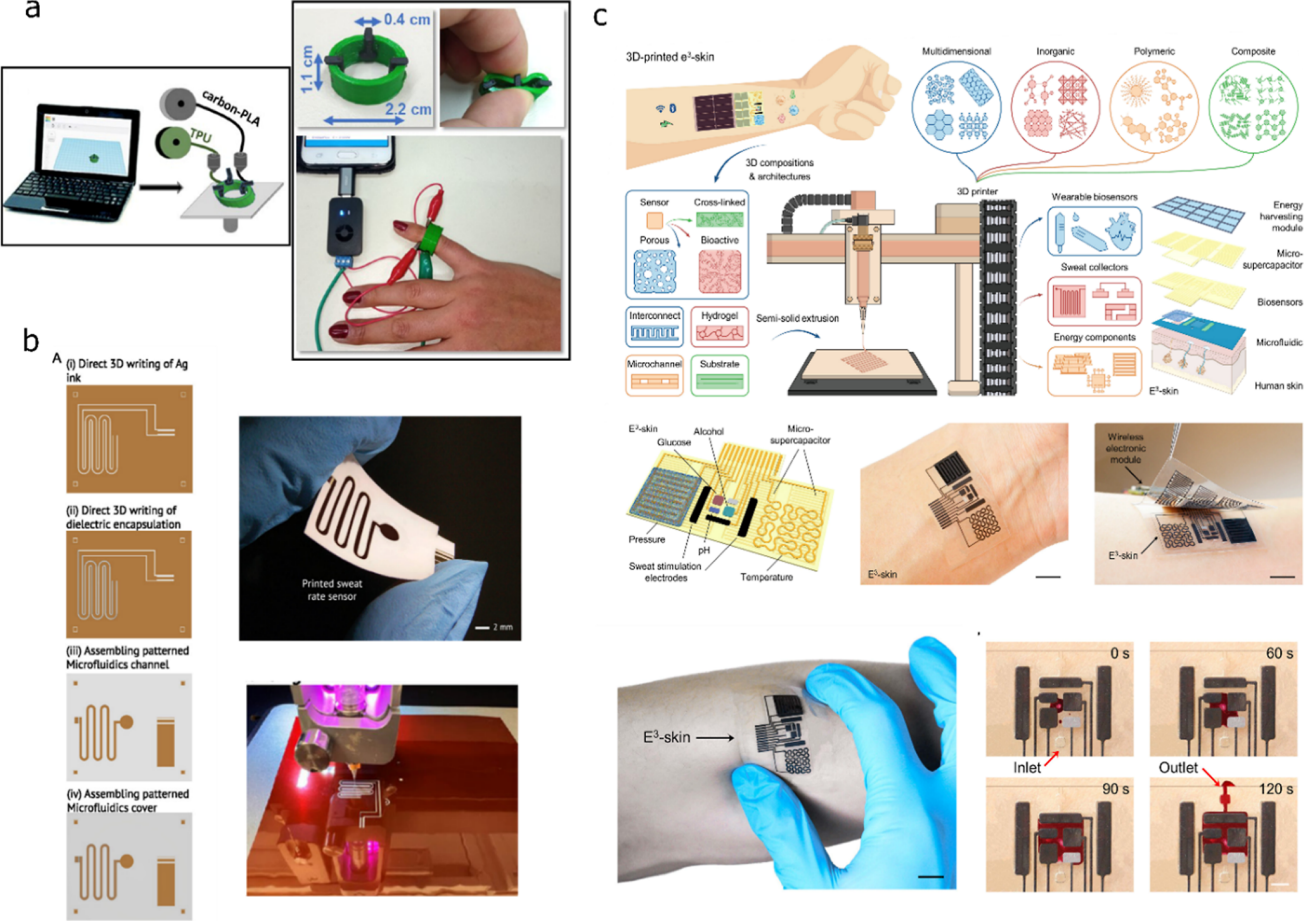
Examples of electrochemical AM epidermal wearable sweat devices found in the literature. (a) Proof of concept of a wearable ring using FFF incorporating nonenzymatic glucose sensing on sweat. Reprinted with permission from ref 58. Copyright 2021, American Chemical Society. (b) DIW schematics and printing process of a sweat rate device. Reprinted with permission from ref 59. Copyright 2024 John Wiley& Sons. (c) Scheme and real pictures of e-skin fully 3D-printed using DIW. Reprinted with permission from ref 60. Copyright 2023, The American Association for the Advancement of Science.

In another direction, some works have incorporated complex electronic sensors in addition to microfluidics into wearable devices. Islam et al. used DIW to realize a capacitive sweat rate sensor.^59^ It is constructed on a flexible polyimide substrate with printed silver electrodes and a dielectric layer to prevent direct contact with sweat. Figure 8b shows the printing and fabrication process of the sweat-rate device. The microfluidic channel is created using patterned double-sided tape, and its volume can be adjusted by changing its design or stacking additional layers. Therefore, in this case, while 3D printing provides a solution for the electrodes and insulator, it has not been proven to print the microfluidic channels, since manual steps are still required to assemble the whole device.

From our point of view, one of the most advanced 3D-printed wearable sensors has been recently reported by the Gao group.^60^ Figure 8c shows the e-skin fabricated using DIW, including several components such as physical and biochemical sensors, microfluidic channels for sweat sampling, and a supercapacitor for energy management. All these elements were 3D-printed, specifically including glucose, alcohol, and pH sensors, as well as temperature and strain sensors. The 3D-printable materials for building the sensors were custom-made, and the compounding of the materials was precisely controlled to enable their printability. Yet, despite the impressive advancement, assembly steps are required to join the different layers, preventing the full automation of the fabrication workflow.

## Outlook for Achieving the “Click-and-Run” Concept

A decade ago, it was challenging to predict that almost every laboratory would have a 3D printer by 2025, as is currently the case. Considering that learning experience, how feasible and how many years it will take to produce a wearable sensor with the “click-and-run” workflow? We do not have a sharp answer, but it is highly probable that it will be feasible in less than ten years. Nevertheless, we want to make it clear that even in that scenario, 3D printing will likely not supplant traditional fabrication methods such as laser cutting or hot-embossing for microfluidics, screen printing, laser-induced graphene, or inkjet printing in the production of planar electrodes. We understand that the potential of 3D-printed electrochemical sensors is boosted when they are combined into a single device, including several materials and complex geometries.

Currently, it is essential for researchers involved with 3D-printed sensors to have a thorough comprehension of the 3D printing process and the influence of printing parameters on the analytical performance of the produced wearable sensors. Shergill and Rocha illustrated the impact of several printing factors, including printing speed, temperature, orientation, and nozzle diameter, on electrode performance.^61,62^ Nonetheless, AI is presently being integrated with highly promising outcomes in material identification and automation of the entire printing process.^63^ Thus, recent advancements have demonstrated the use of AI in enhancing design, detecting defects in real-time during printing, and forecasting component quality.^64^ We envision that the incorporation of AI will advance AM by providing the necessary tools to facilitate “click-and-run” functionality, allowing for optimal selection of material combinations, printing parameters, and recommendations for postprocessing modifications based on the intended analyte or application. A user may input a specified detection target and obtain a fully tuned, functional printed sensor with minimal human intervention.

Aside from the 3D printing process itself, we found out that the 3D printing methods discussed above display some important bottlenecks impeding the meeting of all requirements for the “click-and-run” fabrication of a wearable electrochemical device. In the following paragraphs, we reflect upon their limitations to print each of the device parts.

(1) **Electrodes.** One of the most important constraints for printing conductive tracks is the resulting poor conductivity shown by FFF (0.058 S cm^–1^) and VPP techniques (0.857 S cm^–1^).^65,66^ By contrast, using techniques such as DIW or AJP, much higher conductivity can be obtained ranging from 8 to 10^5^ S cm^–1^. The conductivity levels are adjustable with a proper ink formulation.^67–69^ Nevertheless, the poor conductivity showed with FFF can also be mitigated using distinct approaches. For example, geometric constraints can be added, such as shortening the electrode length resulting in a decrease of the overall resistance.^70,71^ In this regard, Veloso et al. presented empirical evidence on the influence of such resistance on the voltammetric profile; thus, this consideration is crucial when developing the wearable sensor.^47^ Therefore, AJP and DIW are clear candidates for the printing of conductive tracks. Also, electrodes to induce sweat secretion can be printed using DIW. Indeed, iontophoresis system has been fully 3D-printed by the Gao group using DIW.^60^ In addition, Joule heaters have been 3D-printed and can find applications for sweat induction.^72^ Unfortunately, conductivity is not the sole issue. In the common FFF, electrodes must be activated for many sensing applications. Activation is often performed through electrochemical treatment, electrode polishing, and solvent immersion.^73^ Recently, laser ablation of the surface has shown to be a solution more directly compatible with the automation.^74–76^ Indeed, the integration of a laser ablation toolhead with an FFF printing head can yield highly electroactive 3D-printed electrodes. AJP has also been demonstrated to be effective for the creation of electrodes, so it can also operate as a printing head for electrode fabrication.
(2) **Microfluidics and inert components.** We selected FFF as the optimal method for fabricating sub-100 μm microfluidic channels, offering sufficient resolution for sweat epidermal wearable devices. The dimensions of the channels that can be potentially printed allow us to collect and measure sweat samples even in instances of passive sweating, where sweat rates and volumes are minimal.^46^ For example, a 100 × 100 μm channel, printable using FFF, with a flow rate of 10 nL/min, typical of passive sweating, will be filled in approximately 5 min along a length of 5 mm. In other sweating settings (stimulated or active), the flow rate will be boosted while the filling volume will be reduced. In order to apply the sweat sensors in passive sweating scenarios, the pressure generated by the sweat glands is not enough to fill the channel. Therefore, osmotic sweat extraction systems are required for sweat sampling, as it will be discussed below.^77^ In terms of channel resolution, DLP excels in channel resolution; nonetheless, it is unsuitable for multimaterial printing.^78^ DIW has commendable resolution; however, it requires a soluble support material (e.g., PVA, PVP, salt-based), unlike FFF. Quero et al. demonstrated the feasibility of fabricating microfluidic channels with dimensions < 100 μm with desktop 3D printers through a comprehensive understanding of the impact of printing parameters on the obtained resolution.^79^ Nelson et al. demonstrated the feasibility of fabricating sub-100 μm channels using TPU, a highly sought-after flexible material for the production of wearable sensors.^80^ This capability of processing flexible materials enables FFF to build direct interfaces with skin without the necessity of tape for sealing the patch.^54^ To the best of our knowledge, no current reports exist about the 3D printing of a sweat extraction device for passive sweating monitoring via osmotic extraction. Nonetheless, the formulations of the utilized gels documented in the literature are entirely compatible with DIW printing.^81–84^

(3) **The (bio)­recognition elements** are the most delicate components of the (bio)­sensor due to their inherent temperature and chemical sensitivity. Thus, they may limit the selection of the 3D printing technique(s). In principle, FFF is not the most adequate choice because of the high temperatures required to melt the thermoplastic material. In fact, incorporating chemical recognition elements into 3D-printed parts is a challenge itself, and most publications on the subject only addressed 3D printing of conductive substrates for further enzyme immobilization in postprocessing steps rather than direct printing.^85^ DLP has been demonstrated to be a valuable method for the fabrication of membranes incorporating (bio)­recognition elements, such as ionophores,^36^ enzymes,^53^ and antibodies trapped in membranes, based on acrylate-based resins.^86^ However, the postprocessing steps and the challenges associated to multimaterial printing make it difficult to integrate this technique in the “click-and-run” option. From our point of view, DIW stands out as the most convenient technique, owing to its proven ability to print different materials, e.g., hydrogels, silicones, and photocurable polymers. These materials can act as matrix for the physical entrapment of (bio)­recognition elements, including enzymes, aptamers, antibodies, or ionophores. Among some examples, DIW was used to print photocurable alginate hydrogels containing laccase.^87^ Also porous membranes containing β-galactosidase could be printed.^88^ It is important to highlight that the materials compatible with DLP can be translated to DIW, since the photocuring mechanism is compatible with the DIW technique.

Another important consideration when designing wearable patches is the mechanical property optimization for comfortable wearability of the patches. For this purpose, a low bending stiffness is required, being this achieved by using thin substrates with low Young’s modulus (<100 MPa). A good stretchability (>30%) is usually also required.^89^ For instance, flexible microfluidics fabricated with FFF and using TPU as material showed a Young’s modulus of ∼50 MPa and an elongation at break of ∼350%.^80^ Using DIW, the combination of soft inert materials and conductive materials has been also reported, with elongations up to 100% without losing conductivity performance.^90^ Another recent trend interesting for wearable fabrication is the development of 3D-printable skin bioadhesives. Certainly, bioadhesives have demonstrated the possibility of being printed by DIW^91^ and hybrid printing combining SLA and DIW in two printing steps.^92^ Additionally, it has recently been reported that conductive soft materials based on PEDOT/PSS printed with DIW showed a Young’s modulus of 0.75 MPa and high printing resolution (∼50 μm).^93^

It is important to highlight the importance of considering material biocompatibility during the selection process. Devices must meet the requirements of the biocompatibility requirements ISO 10993 for medical devices being nonirritating for skin, noncytotoxic, hemocompatible, and being nondegraded or degraded into safe substances during skin contact.^94^ Most of the materials used in FFF and DIW are biocompatible and even medical-grade commercial filaments can be purchased with proven biocompatibility.^95,96^ Also, DLP resins can be considered biocompatible after certain postprocessing steps.^97^

Accordingly, we believe that a holistic solution for the “click-and-run” printing of wearable devices must rely on hybrid printing (i.e., combination of different printing techniques in the same printer). It is important to note that the “click-and-run” approach goes beyond the state-of-the-art, since it will enable the complete automation of the fabrication process using the most appropriate printing technique for each sensor component. Figure 9 illustrates the proposed design of an ideal 3D printer wherein the combination of different techniques will enable the full printing of any wearable device. It contains several toolheads that enable to fulfill the specifications for each component and layer in the wearable sensor architecture. A closed-loop 3D printing process will be implemented in the printer; therefore, the different layers of the selected prototype will be printed sequentially with the most appropriated printing head to produce the full assembled wearable. Moreover, we envision that the wearable field will begin to embrace customized hybrid 3D printers in the next years to address the challenge of “click-and-run” printing for wearable devices.

**Figure 9:**
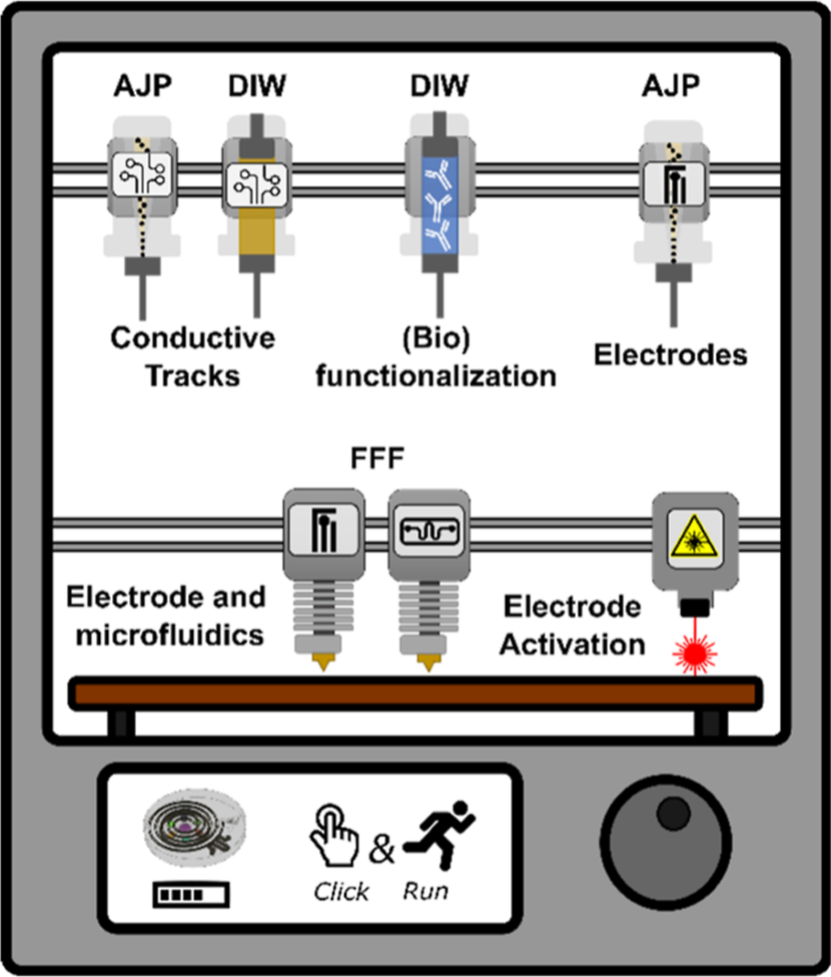
Proposed hybrid 3D printer incorporating the different AM techniques with the best characteristics for printing each of the epidermal wearable sensor patches. Toward “click-and-run” concept.

Recently, Roach et al. have proposed a multimaterial and multimethod 3D printer (m4 3D printer), incorporating IJ, FFF, DIW, and AJP, along with robotic arms for pick-and-place (PnP).^98^ The m4 printer demonstrated the capability to fabricate both insulating (FFF) and conductive materials (DIW) in addition to incorporating electronic components to fabricate an LED light seamlessly (PnP). We strongly believe that this multitool and multimaterial capability, which has been specifically designed and adapted for the fabrication of wearable electrochemical sensors such as the one proposed in Figure 9, will revolutionize the field of electrochemical wearable sensors in the near future. Indeed, recent examples of hybrid printing with applications in the (bio)­sensing domain support our vision about the implementation of hybrid printing. In such a context, Due et al. coupled direct ink writing (DIW) with aerosol jet printing (AJP) to develop a lactate enzyme biosensor within a microfluidic device.^99^ This innovative technology, although groundbreaking, requires more refinement to achieve the “click-and-run” paradigm, as support materials are necessary to produce microfluidics. This necessitates postprocessing processes, and it remains ambiguous when the alteration of the functioning electrodes occurs in an automated way. Zhang et al. presented another example of hybrid printer solutions, not utilized for chemical sensing, which involved the integration of liquid dispensing and laser treatment of FFF surfaces within a single 3D printer.^100^ Truly, this opens the possibility of embedding highly conductive tracks and electrodes owing to the synergy between FFF, laser engraving, and liquid dispensing within a customized printer. Laser engraving was used to create highly conductive graphene tracks on 3D-printed polycarbonate pieces instead of using the classical polyimide substrates. Additionally, the printer can dispense the liquid precursors to create metallic tracts by dispensing the precursor over the laser-engraved carbon tracks, which can be further laser treated.

It is important to emphasize the advantages of AM in facilitating the introduction of prototypes into the market. The transition of laboratory devices to mass manufacturing continues to pose an unresolved difficulty, even with the application of traditional fabrication processes, such as laser engraving or screen printing. Nonetheless, AM advantages can leverage the introduction of wearable devices designed in academic settings into practical, real-world applications in a more efficient and streamlined manner.

It is important to emphasize that the integration of AI may be a more long-term objective for the complete automation of the printing process (steps I–IV in Figure 1). However, the automation of production steps II and IV can be achieved in the short term by combining the knowledge derived from hybrid 3D printing hardware and wearable sensing knowledge.

Finally, we need to move beyond traditional paradigms and harness the potential of 3D printers to advance the field of wearable technology. If this becomes a reality, the disruptive materialization of the “click-and-run” printing concept will soon blur the line between traditional and additive manufacturing.
